# Comprehensive review of multidimensional biomarkers in the ShangHai At Risk for Psychosis (SHARP) program for early psychosis identification

**DOI:** 10.1002/pcn5.152

**Published:** 2023-11-14

**Authors:** TianHong Zhang, LiHua Xu, XiaoChen Tang, YanYan Wei, YeGang Hu, HuiRu Cui, YingYing Tang, ChunBo Li, JiJun Wang

**Affiliations:** ^1^ Shanghai Engineering Research Center of Intelligent Psychological Evaluation and Intervention, Shanghai Key Laboratory of Psychotic Disorders, Shanghai Mental Health Center Shanghai Jiaotong University School of Medicine Shanghai China; ^2^ Shanghai Key Laboratory of Psychotic Disorders, Shanghai Mental Health Center Shanghai Jiaotong University School of Medicine Shanghai China; ^3^ CAS Center for Excellence in Brain Science and Intelligence Technology (CEBSIT) Chinese Academy of Sciences Shanghai China; ^4^ Institute of Psychology and Behavioral Science Shanghai Jiaotong University Shanghai China

**Keywords:** biomarkers, clinical high risk, prediction, psychosis

## Abstract

Psychosis is recognized as one of the largest contributors to nonfatal health loss, and early identification can largely improve routine clinical activity by predicting the psychotic course and guiding treatment. Clinicians have used the clinical high‐risk for psychosis (CHR) paradigm to better understand the risk factors that contribute to the onset of psychotic disorders. Clinical factors have been widely applied to calculate the individualized risks for conversion to psychosis 1–2 years later. However, there is still a dearth of valid biomarkers to predict psychosis. Biomarkers, in the context of this paper, refer to measurable biological indicators that can provide valuable information about the early identification of individuals at risk for psychosis. The aim of this paper is to critically review studies assessing CHR and suggest possible biomarkers for application of prediction. We summarized the studies on biomarkers derived from the findings of the ShangHai at Risk for Psychosis (SHARP) program, including those that are considered to have the most potential. This comprehensive review was conducted based on expert opinions within the SHARP research team, and the selection of studies and results presented in this paper reflects the collective expertise of the team in the field of early psychosis identification. The three dimensions with potential candidates include neuroimaging dimension of brain structure and function, electrophysiological dimension of event‐related potentials (ERPs), and immune dimension of inflammatory cytokines and complement proteins, which proved to be useful in supporting the prediction of psychosis from the CHR state. We suggest that these three dimensions could be useful as risk biomarkers for treatment optimization. In the future, when available for the integration of multiple dimensions, clinicians may be able to obtain a comprehensive report with detailed information of psychosis risk and specific indications about preferred prevention.

## INTRODUCTION

Psychosis, represented by schizophrenia, is not only a clinical problem in psychiatry but also a major challenge in brain science research. However, it is also an unavoidable problem that concerns China's 16 million patients, as well as people's livelihoods and civil affairs, and social and family harmony. The characteristics of the course of psychosis are very prominent; that is, nearly 50% of the patients will enter a chronic and irreversible process once the onset of psychosis occurs, which is the eighth most common reason for the loss of disability‐adjusted life years for people aged 15–34 years worldwide. This clinical outcome further emphasizes the importance of early identification and treatment in this population.

Internationally, a general consensus has been reached regarding the clinical criteria for the “at‐risk” state of psychosis. In 1927,^1^ scientists proposed that the traditional treatment of schizophrenia was administered too late. Although many patients show nonspecific clinical symptoms before onset, clinicians often deal with symptoms in the late stage of the disease. In other words, psychosis treatment may have missed the best intervention window. In the past 30 years, scientists have focused on the clear concept of the clinically high risk for psychosis (CHR).^2^ People with CHR generally have attenuated positive symptoms of psychosis, such as hallucinations or delusions, but the degree is relatively low (with insight into these symptoms), and the course of disease is short (generally no more than 1 year). Three key points in this definition indicate that individuals with CHR are the most suitable population for early identification. The first feature is positive symptoms. In contrast to negative symptoms and general nonspecific symptoms, positive symptoms have the highest clinical recognition and the best operability in clinical recognition, which makes it feasible to clinically recognize people with CHR status. The second feature is insight, which is the core key for CHR to distinguish the first episode of psychosis, and it is precisely this feature that makes it more urgent for the CHR population to seek medical help than the patients after the disease onset. Insight is a prominent factor that frequently emerges in the clinical context.^3^, ^4^ Third, while CHR states can sometimes have a short duration and acute characteristics, they can also extend over 3–5 years, making them more visible, identifiable, and feasible for clinical practice in certain cases.

## CLINICAL PREDICTION OF PSYCHOSIS

The CHR status is recognizable, and the possibility of conversion to psychosis in this group in the next 1–3 years is much higher than that of the general population. It is generally accepted that this proportion is approximately one‐third. Poli et al.^5^ conducted a meta‐analysis of the clinical outcomes of 2502 individuals with CHR (from 1996 to 2011). The average conversion rate to psychotic attack was 29.2% in the 31‐month follow‐up period, and 17.7%, 21.7%, 26.9%, 29.1%, and 31.5% in each time node of 6, 12, 18, 24, and 36 months, respectively. In addition to the meta‐analysis of perspective of long‐term outcome, Nelson et al.^6^ reported that until the end of the 10th year, there were still risks remaining for psychosis recurrence. Compared to the approximate prevalence rate of psychosis of about 4.62 per 1000 in the general Chinese population,^7^ individuals at a genetic risk stage were associated with a nearly 10‐fold higher risk of developing psychosis.^8^ CHR individuals can be more effectively identified, showcasing a significantly higher incidence rate and this holds immense clinical importance in the accurate identification of CHR status.

## BIOLOGICAL MARKERS

By reviewing CHR studies on biological‐marker‐related predictions, there has been increasing evidence of significant changes in the biological characteristics of the CHR population identified clinically. In the cohort, there were significant differences between the CHR converter (CHR‐C) group (converted into the first episode of psychosis) and the nonconverter (CHR‐NC) group in a number of neurobiological indicators, including brain volume, cortex thickness, white matter integrity, brain functional connectivity, and inflammation level. For example, the North American Prodrome Longitudinal Study (NAPLS) multicenter research suggests that CHR with reduced gray matter volume in the right frontal lobe and enlarged third ventricle has a higher risk of conversion to psychosis.^9^ A translocator‐protein positron emission tomography (PET) imaging study found that microglial cells were also overactivated in CHR, which was related to the severity of symptoms, suggesting that the increased level of neuroinflammation was related to the onset of psychosis.^10^, ^11^, ^12^ The Outreach and Support in South London (OASIS) study found that the integrity damage of left frontal lobe white matter in the CHR‐C group was more serious than that in the CHR‐NC group.^13^

## SHANGHAI AT RISK FOR PSYCHOSIS (SHARP)

The ShangHai At Risk for Psychosis (SHARP) team was established at the Shanghai Mental Health Center in 2010. Through in‐depth cooperation with the Harvard team led by Professor Larry Seidman of the Harvard Medical School, a series of studies on the clinical and biological recognition of CHR have been carried out.

First, the SHARP team took the lead in formulating the concept and diagnostic criteria for CHR that were suitable for China's cultural background. While the diagnostic criteria for CHR in China remain essentially consistent with international standards, the emphasis was placed on ensuring that the terminology and wording used in the Chinese version were culturally relevant and aligned with China's cultural context. Screening and clinical diagnostic tools were also developed to facilitate the identification of individuals at CHR status in the Chinese population.^14^, ^15^ Among the help‐seeking populations in professional mental health institutions, it was first reported that the positive detection rate of CHR was 4.2%, with an average age of 21 years, which was highly consistent with the data of foreign cohorts through longitudinal follow‐up verification.^16^, ^17^ To our knowledge, this is the first time that Chinese scientists have successfully identified high‐risk clinical groups. The SHARP team trained more than 1000 domestic psychiatrists through a series of courses, covering most of the provinces and cities in China. Consequently, they significantly improved the identification rate of psychotic risk in China, and have been widely recognized by their domestic peers. In 2019, an expert consensus was formed on the early identification and intervention of CHR in China. This is the first and only expert consensus on such topics in China, which is a milestone.

The SHARP team aimed to build a high‐quality cohort, study relevant risk factors for psychosis, assess the pathogenesis, and develop new prevention and control measures.^18^, ^19^, ^20^, ^21^ By the end of 2022, there were more than 1600 clinical high‐risk patients in the SHARP cohort. The average follow‐up time was 5.5 years, the longest follow‐up time was nearly 12 years, and the rate of lost follow‐up of the entire cohort was <15%. The SHARP cohort comprehensively provides 10 dimensions of behavioral and biological information of high‐risk patients, including clinical, cognitive, brain imaging (including magnetic resonance, EEG signal, and near‐infrared brain function signal), blood biochemical metabolism, genetics, immune response, and eye movement trajectory. This is the largest single‐center recruitment cohort with long‐term follow‐up time and complete high‐quality biological markers worldwide.

Next, we will describe the three dimensions of biomarkers found in the SHARP cohort that have important value in the early warning of psychosis. In the SHARP project, a single MRI scanner is used, specifically the Siemens 3T MR B17 (Verio) system, equipped with a Siemens 32‐channel head coil. For the other dimensions of biomarkers, event‐related potentials (ERPs) data are collected using a 64‐channel EEG system with surface electrodes mounted in an elastic cap (EasyCap; Brain Products Inc.). Immunology assessments involve drawing 10 mL of peripheral venous blood into anticoagulant‐free tubes. In total, among the 1600 CHR individuals enrolled in the study, approximately 80% of them completed data collection for the three dimensions of biomarkers. Table 1 summarizes the relevant literature from the SHARP study that was reviewed in this article. It presents the sample sizes of CHR individuals, their demographic characteristics, and the major findings related to biomarkers.

**Table 1:** Characteristics of studies included in the review.

Study	CHR			Biomarkers
	*N*	Male	Age	
*Neuroimaging dimension of brain structure and function*				
Collin et al.^22^	158	80	19	Abnormal modular connectome organization, including superior temporal gyrus and anterior cingulate cortex
Tang et al.^23^	50	30	20	Free‐water imaging white matter measures: fractional anisotropy of cellular tissue alterations
Anteraper et al.^24^	144	73	19	Resting‐state functional connectivity analysis: abnormal function in dentate nuclei
Del Re et al.^25^	152	76	19	Cortical thickness measures: frontotemporoparietal abnormalities
*Electrophysiological dimension of event‐related potential*				
Wu et al.^26^	92	39	19	Mismatch negativity (MMN): reduced frequency MMN (fMMN) and duration MMN (dMMN)
Tang et al.^23^	104	55	19	Auditory P300 oddball and novel components: reduced P300 novel amplitude
Wu et al.^33^	104	53	18	ERP and time–frequency: reduced P300 response, decreased delta event‐related spectral perturbation and intertrial coherence
*Immune dimension of inflammatory cytokines and complement proteins*				
Zhang et al.^27^	84	49	19	Th1/Th2 imbalance: reduced serum IL‐1β level, decreased IL‐1β/IL‐6 ratios
Zhang et al.^28^	37	19	18	Th1/Th2 imbalance: decreased IL‐2/IL‐6 ratios
Zhang et al.^29^	394	192	19	Inflammatory markers: IL‐1β, IL‐2
Gan et al.^30^	105	46	18	Attenuated niacin‐induced flushing
Zhang et al.^31^	49	35	18	Serum complement proteins: reduced levels of C5 and C5a

### Neuroimaging dimension of brain structure and function

Changes in brain structure and function in the CHR state of psychosis are relatively direct biomarkers for the early prediction of the first episode. Patients with schizophrenia showed a significant reduction in cortical thickness. The SHARP team^25^ then determined whether the cortical thickness (CT) and surface area (SA) of the frontal lobe, temporal lobe, and parietal lobe in the CHR cohort were different from those in the healthy control (HC) group. The results showed that the CT, rather than the SA, in the CHR population was significantly reduced compared with HCs. We found two patterns: (1) compared with the HC and CHR‐NC groups, the thickness of the banks of the superior temporal sulcus, Heschl's gyrus, and pars triangularis in the CHR‐C group was significantly reduced; and (2) compared with the HC group, the thickness in the inferior parietal and supramarginal gyrus, and at the trend level in the pars opercularis, fusiform, and middle temporal gyri was significantly decreased in all individuals with CHR to varying degrees. In addition, reduced CT was significantly related to the age of HCs and the CHR‐NC group, but this correlation was not observed in the CHR‐C group.

In the initial stages of psychosis, studies have shown that both intracellular and extracellular white matter changes occur simultaneously. The SHARP team studied^23^ the changes in intracellular and extracellular white matter in individuals with CHR who had never been treated with psychotic drugs. The SHARP team used the advanced diffusion tensor imaging (DTI) model of free water (FW) imaging to improve the specificity of white matter damage in CHR and achieved a fine distinction between the role of intracellular white matter damage and extracellular free water enhancement in the CHR stage. In our innovative DTI technology, the conventional single‐tensor model is replaced by a double‐tensor model. This approach considers multiple factors influencing the fractional anisotropy (FA) of water molecules in brain tissue, including axon diameter changes, demyelination, extracellular component variations, and axon tissue alterations. Conventional DTI‐based FA indices lack specificity, but our method distinguishes between intracellular FA (FAT) and extracellular FA (FW), with the extracellular FW fraction primarily indicating inflammation. At the overall level of the white matter skeleton, the routine FA value of the CHR population was lower than that of the HCs. The free water model analysis further suggests that the low FA value of the CHR population is mainly due to the decrease in the intracellular FAT value, while the extracellular FW component showed no significant change. There was a significant interaction between group and age for the FAT value. The FAT value in the HC group increased with age, but there was no significant correlation between the FAT value and age in the CHR group, which was significantly correlated with a decline in general function. At the voxel level, it was observed that the routine FA and FAT values of the CHR population decreased, and the distribution was mainly in the corpus callosum, right corona radiata, and bilateral superior longitudinal tract; the FW value had no obvious abnormality. It suggested that abnormal white matter integrity already existed in the CHR stage.

To study whether abnormalities in connectome organization precede psychosis onset, on a subset of 158 CHR individuals, the SHARP team conducted a large‐sample resting‐state functional connectivity (RsFc) analysis. Compared with CHR‐NC individuals, CHR‐C individuals showed abnormal modular connectome organization at baseline. The analysis of brain connectivity based on regions showed that the brain regions with abnormalities in the CHR population, including the superior temporal gyrus and anterior cingulate cortex, were the most abnormal in terms of modular assignment. The results showed that functional changes in brain network organization precede the onset of psychosis and may promote the development of adolescents at risk for the clinical outcome of psychosis.^22^ The project team used the connectomics method to study the brain function of the CHR population and introduced the graph theory to analyze the brain structure and functional connectivity networks. The characteristics of connectomics indicate that the brain network has the characteristics of a modular organization, which can be divided into highly connected modules and relatively low‐connected brain regions. The modular organization model of “superior temporal gyrus sensorimotor region limbic system” of the CHR population can clearly predict the risk of psychosis attack 1 year later, and the probability of psychosis is more than three times higher. The term “modular organization” refers to the grouping of brain regions into distinct modules or communities based on their functional connectivity patterns. These modules represent clusters of brain regions that share similar patterns of connectivity, and our analysis using the Louvain community detection method allowed us to identify these modules within the functional connectome of CHR individuals. This approach provides valuable insights into how different brain regions within these modules interact and contribute to predicting the risk of psychosis onset 1 year later. This feature can be used for the biological prediction of psychosis.

The cerebellum has a wide range of functions and is composed of discrete regions that perform unique functions. The dentate nucleus (DN) is the main output nucleus of the cerebellum. A new segmentation method for the DN optimally divides the structure into three functional territories that uniquely contribute to default mode, motor salience, and visual processing networks, as indexed by RsFc.^24^ Here, we studied for the first time whether the RsFc difference in DN exists in the CHR population, which precedes the onset of psychosis. The RsFc analysis proposed by the research group showed a significant difference between the baseline RsFc values of CHR‐C and CHR‐NC individuals. The changes observed in the brain match the three functional areas of DN very well. The initial detection of abnormal RsFc in DN may precede the onset of psychosis. This new evidence suggests that the cerebellum is a potential target for the prediction and prevention of psychosis. The observed abnormalities in DN functional connectivity provide insights into potential neural mechanisms associated with the development of psychosis. However, the exact nature of how these abnormalities contribute to the pathophysiology of psychosis requires further investigation and may involve a combination of pathological changes and compensatory mechanisms.

### Electrophysiological dimension of ERP

ERP signals can reflect the abnormal state of brain function of subjects to a certain extent. ERP components under different paradigms can effectively reflect the abnormal mechanisms of brain function in patients with psychosis. The SHARP team^26^ systematically studied the characteristics of mismatch negativity (MMN) in the CHR population. The MMN is a widely studied ERP component that reflects the processing of preattention auditory information. Auditory stimuli can be divided into standard and deviant. The deviation stimulus included two types: frequency and duration deviation stimuli (pure tone with different frequencies and durations, respectively, from the standard stimulus). The MMN amplitude was calculated from the average amplitude in the 100–200‐ms window. We found that individuals with CHR had abnormal MMN induced by frequency and duration deviation stimuli. At baseline, the MMN of the CHR‐C group was lower than that of the CHR‐NC group, and the MMN of the CHR‐C group decreased more over time than that of the CHR‐NC group. The MMN responses in our CHR subtypes carry significant implications, as they are linked to clinical factors and offer potential markers for personalized interventions. These MMN indexes exhibit superior predictive capabilities for remission within specific clusters, emphasizing their role as valuable objective markers in guiding CHR clinical management.

An ERP is a potential change that occurs when individuals perform cognitive processing. This process involves the level of human attention, memory, emotion, and other brain functions. The SHARP team^32^, ^33^ found that latency and peak changes of P300, the endogenous component of ERP, were affected to some extent in the CHR population. The oddball paradigm was used to elicit classical P300 components and the novel paradigm was used to elicit novel P300 components. Classical auditory P300 and novel P300 are biomarkers of neurocognitive changes in chronic and first‐episode schizophrenia. Classical P300 is linked to processing task‐relevant or target stimuli, while novel P300 is associated with detecting unexpected, nontarget stimuli in the environment, reflecting distinct cognitive processes and attention mechanisms. The amplitude and latency of the classical and novel P300 were compared among the CHR‐C, CHR‐NC, and HC groups. The results showed that the CHR‐C group had a lower frontal central novel P300 amplitude and a slightly lower classical P300 amplitude than the HC group. The amplitude of novel P300 in individuals with remission CHR was equivalent to that in HCs and higher than that in CHR‐C subjects. Therefore, the reduced amplitude of novel P300 represents a significant feature of individual impairment that can be used as a sign of psychosis conversion.

### Immune dimension of inflammatory cytokines and complement proteins

The occurrence and development of psychosis is not only a change in behavioral appearance or brain function but also a characteristic change in the characteristics of immunoinflammatory markers in the early stage. The SHARP team^29^, ^34^, ^35^ found that the imbalance of inflammation (CD4 + T helper cell [Th] 1/Th2 cytokine imbalance) was related to psychotic attack in the CHR outcome after studying the balance of inflammatory factors in the peripheral serum of the CHR population. The baseline serum Th1 inflammatory factor concentration in the CHR‐C group was significantly reduced, and can be considered a form of immune deactivation, resulting in a lower Th1/Th2 ratio than that in the CHR‐NC and HC groups. These results indicate that specific patterns of Th1/Th2 imbalance are associated with an increased risk of developing psychosis. The data from SHARP suggest that an imbalance of inflammatory cytokines (interleukin [IL]−1β, 2, 6, 8, 10, and tumor necrosis factor‐α [TNF‐α]) may occur in the CHR stage, especially in individuals with CHR‐C. The hypothesis of an inflammatory immune imbalance is closely related to the lipid peroxidation of the cell membrane. The project team used the semiquantitative water‐soluble methyl nicotinate patch method and a laser Doppler flowmeter to detect the skin flushing reaction of niacin in the CHR population, demonstrating that niacin‐insensitive individuals are an effective biological endophenotype of psychosis.^30^, ^36^, ^37^

Inflammation and immune dysfunction have been implicated in various psychotic disorders. Although differences in serum cytokines and complements between individuals with CHR and HCs have been reported, previous studies have investigated inflammatory and complement biomarkers separately; therefore, we did not know which of the two is better at predicting the onset of psychosis. The SHARP team addressed these issues by assessing and comparing a wide range of inflammatory cytokines and complements in individuals at the CHR phase of psychosis and well‐matched HCs to better understand the effects of immune dysfunction on psychosis development.^31^ Our data demonstrate that the serum levels of complement, specifically C5 and C5a, in individuals with CHR are more strongly associated with conversion to psychosis than inflammatory factors. Therefore, alterations in serum complement levels, particularly C5 and C5a, may precede the first episode of psychosis in individuals with CHR. Complement may also be important for developing specific strategies for monitoring and treating early psychosis.

## FUTURE POTENTIAL CLINICAL USE

Most of the above research from the SHARP team is exploratory, and the conclusions point to the differences between groups, but they fail to integrate multidimensional biological characteristics to improve the prediction accuracy. That is, we can learn from clinical risk calculation studies^38^, ^39^ that these biological markers have the potential to integrate and form a multidimensional model and determine the best intervention plan for the patient. Effective intervention measures are often targeted at specific causes rather than at symptomatic treatment. However, a specific etiological hypothesis (such as inflammatory imbalance) with limited single‐dimensional research can only guide whether the treatment measures for this etiological hypothesis are applicable (such as anti‐inflammatory and antioxidant stress).

This is the only way to apply an intelligent multidimensional model to assist in the decision‐making of individualized identification and treatment schemes for the CHR population. (1) The CHR population is highly heterogeneous, with obvious differences in different biological dimensions and clinical outcomes. These characteristics indicate that solutions with a traditional single dimension are extremely inefficient, and underscore the need for a comprehensive approach that considers multiple dimensions of biomarkers associated with psychosis risk. Tailoring preventive strategies based on an individual's unique biomarker profile across various dimensions may lead to more effective clinical outcomes, and machine learning approaches offer promise in identifying these personalized intervention strategies. (2) The SHARP team has conducted a systematic investigation on whether the CHR population is suitable for the use of traditional antipsychotic drugs for prevention in the early stage. The results suggest that antipsychotic drugs should not be used prematurely in the at‐risk population^27^, ^28^, ^40^ as they are only valuable for <15% of the CHR population of a certain subtype^41^, ^42^, ^43^; it is necessary to carry out individualized new intervention technologies for CHR subtypes of different biological dimensions.^3^ Approximately 30% of the CHR population may recover naturally without intervention, and a large proportion of CHR are teenagers. Therefore, at present, the international medical community tends to adopt relatively conservative protective intervention programs for this group, such as cognitive behavioral therapy,^44^ family therapy, and intake of unsaturated fatty acids.^45^ The adopted intervention needs to meet the characteristics of effectiveness, accuracy and mild side‐effects, which have both efficacy and safety on CHR population. Therefore, it is urgent to carry out more accurate personalized diagnosis and treatment based on an intelligent multidimensional model to effectively prevent an individual's first psychotic attack.

## AUTHOR CONTRIBUTIONS

TianHong Zhang and JiJun Wang conceptualized the study, wrote the first draft of manuscript. LiHua Xu, HuiRu Cui, YanYan Wei, YingYing Tang, YeGang Hu and XiaoChen Tang managed the literature searches and edited the manuscript. ChunBo Li, TianHong Zhang and JiJun Wang designed the study and provided supervision in the implementation of the study.

## AUTHORSHIP STATEMENT

TianHong Zhang, YingYing Tang, ChunBo Li, and JiJun Wang proposed the topic and main idea. XiaoChen Tang, LiHua Xu and YanYan Wei were responsible for the literature search and study selection. YeGang Hu and HuiRu Cui were responsible for the quality assessment. TianHong Zhang wrote the initial draft.

## DISCLOSURE STATEMENT

Jijun Wang is an Editorial Board member of *Psychiatry and Clinical Neurosciences Reports* and a co‐author of this article. To minimize bias, they were excluded from all editorial decision‐making related to the acceptance of this article for publication.

## CONFLICT OF INTEREST STATEMENT

The authors declare no conflicts of interest.

## ETHICS APPROVAL STATEMENT

N/A

## PATIENT CONSENT STATEMENT

N/A

## CLINICAL TRIAL REGISTRATION

N/A

## Data Availability

N/A
